# Supplementary figures and images for: Recurrent mutations in the stress regulator Cap1 reveal a trade-off between azole resistance and oxidative stress response in Candida albicans
Source: PLoS Biol. 2026 Feb 2;24(2):e3003631. doi: 10.1371/journal.pbio.3003631 (PMC12904577; doi:10.1371/journal.pbio.3003631)

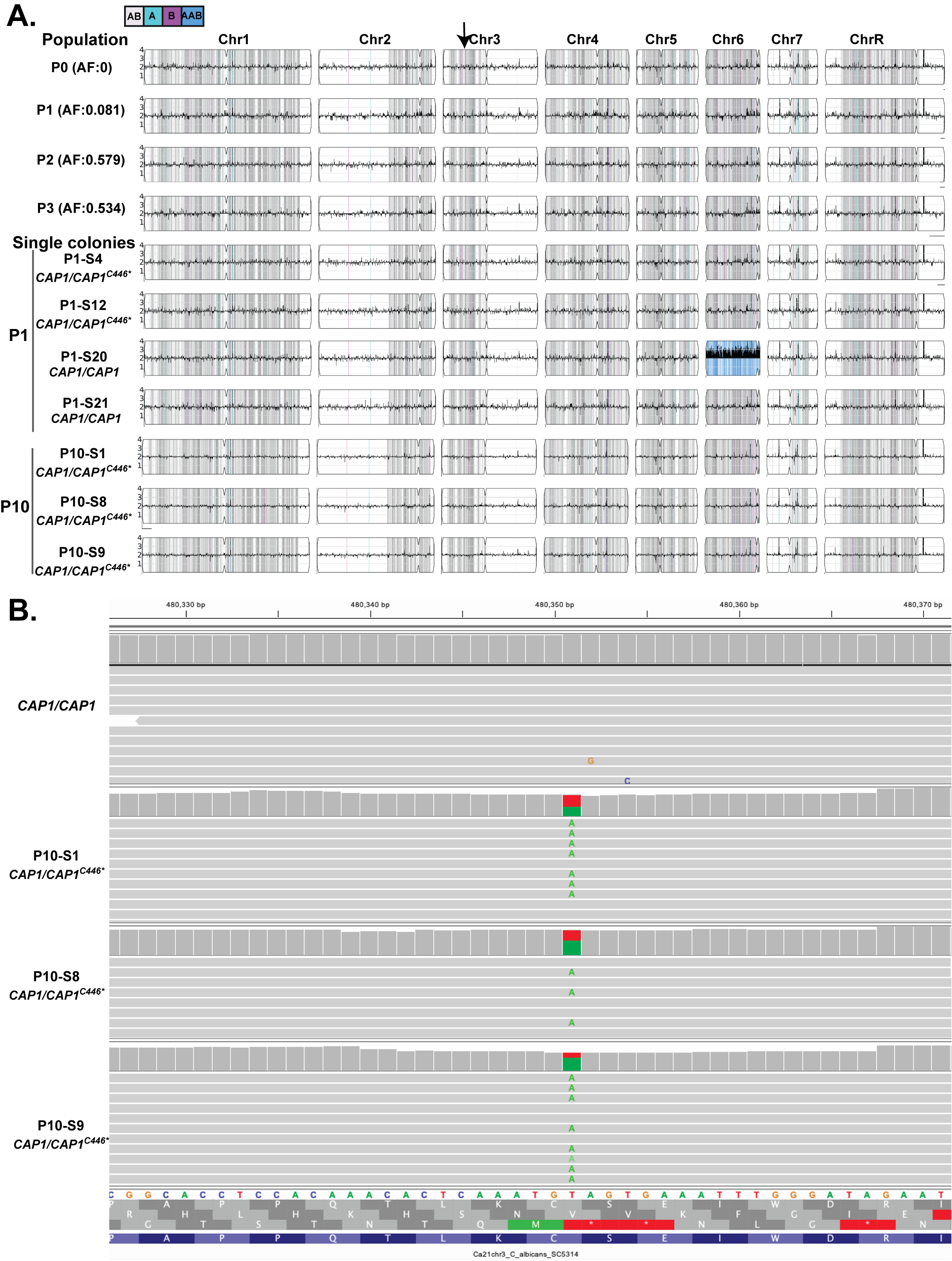

Supplement: S1 Fig — whole-genome sequence data for the entire population at P0, P1, P2, and P3 and seven single colonies from P1 or P10. Data are plotted as the log2 ratio and converted to chromosome copy number (Y-axis, 1–4 copies) as a function of chromosome position (X-axis, Chr1–ChrR) using YMAP [76]. Arrow indicates the position of the CAP1 locus on Chr3. Haplotypes relative to SC5314 are indicated: white is homozygous for the reference strain, grey is heterozygous AB, magenta is homozygous B, cyan is homozygous A, and blue is trisomy AAB. B. A subset of whole-genome sequencing reads mapped to the CAP1 gene (Chr3 positions 480,330–480,370) for the wild-type progenitor (top) and three fluconazole-evolved single colonies from passage 10 (P10). Reads visualized using IGV [77], where gray shading indicates exact alignment to the reference genome. The T-to-A mutation in the three single colonies has an allele frequency of ~0.5 (red = reference base; green = mutant base). The mutation changes a cysteine codon (TGT) to a stop codon (TGA) at amino acid 446. (TIF) [file pbio.3003631.s001.tif]

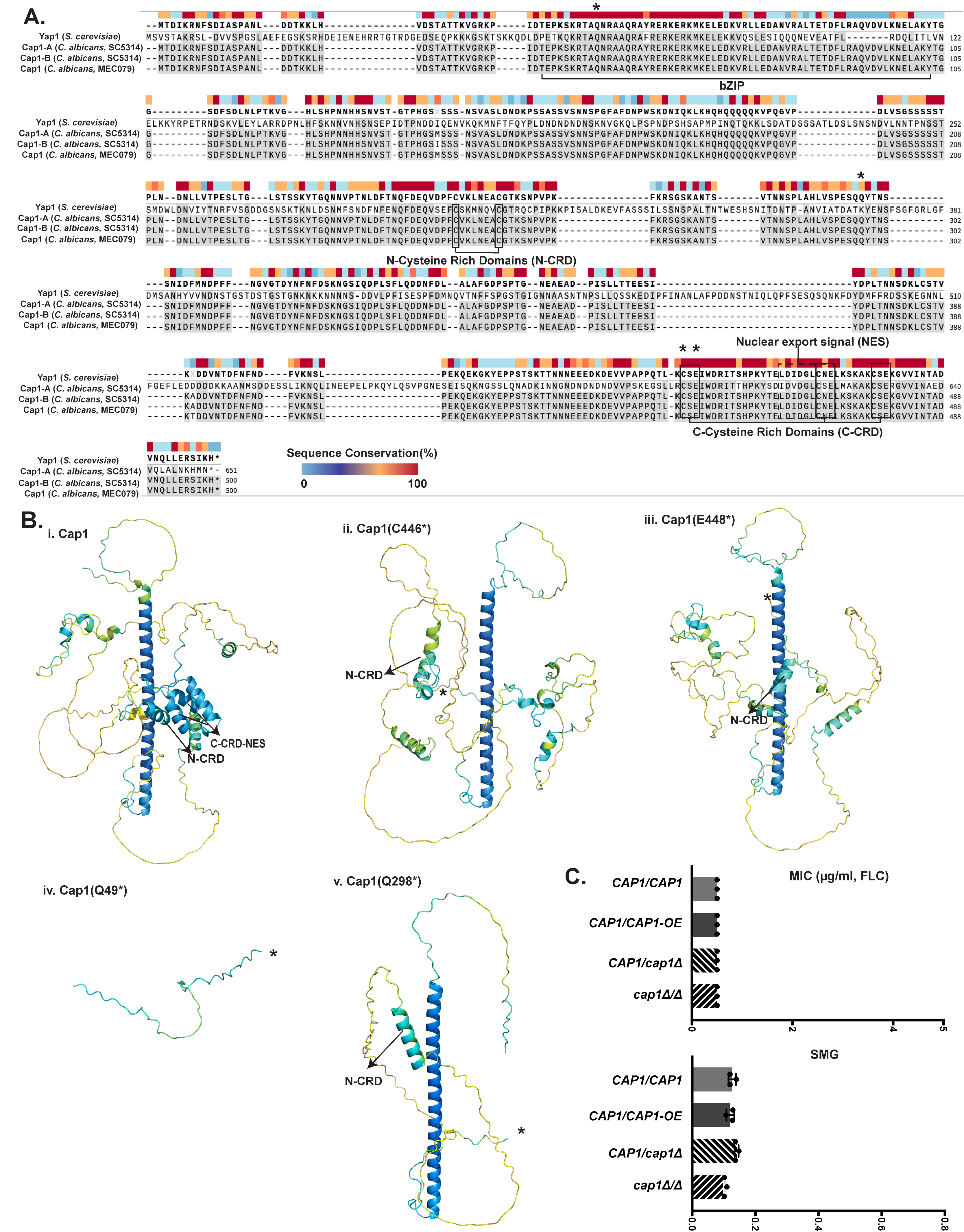

Supplement: S2 Fig — A. Multiple sequence alignment of S. cerevisiae Yap1, C. albicans Cap1-A and Cap1-B from the diploid reference strain (SC5314), and Cap1 from clinical isolate MEC079. Similarity highlighted with gray. Sequence conservation relative to S. cerevisiae Yap1 is indicated with the heatmap (red is 100% conservation). Asterisks (*) indicate the amino acid position where nonsense variants were identified in in vitro and clinical isolates. Black boxes indicate the Cap1 Cysteine Rich Domains (CRD) at the N-terminus (N-CRD) and C-terminus (C-CRD). B. AlphaFold predicted protein structures of wild-type Cap1 (i), and truncated Cap1 with either C446* (ii), E448* (iii), Q49* (iv), or Q298* (v) variants. C. 24 h MIC (top, μg/ml) and 48 h SMG (bottom) in FLC for CAP1 heterozygous overexpressed strain (CAP1/CAP1-OE) and CAP1 heterozygous and homozygous deletion mutants (CAP1/cap1Δ and cap1Δ/Δ) with wild-type strain (SC5314_CAP1/CAP1) as the control. For MIC values, each dot represents a single replicate, and each bar represents the average of three biological replicates of a single strain; SMG values are mean ± SD calculated from three biological replicates of a single strain. The data underlying this Figure can be found in https://doi.org/10.5281/zenodo.18250101. (TIF) [file pbio.3003631.s002.tif]

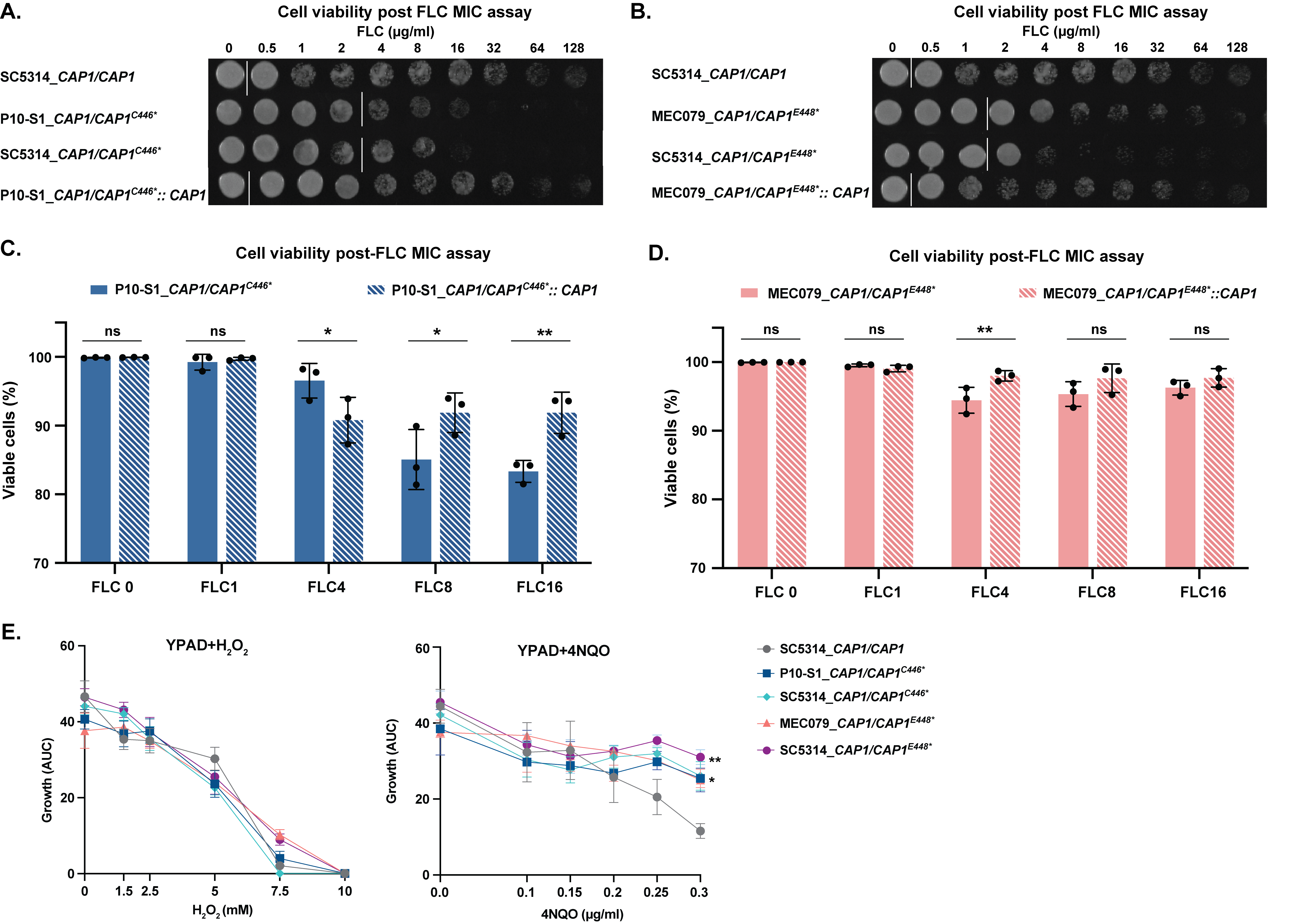

Supplement: S3 Fig — A&B. Cells from the 48 h MIC assay in Fig 2B, were plated for viability on YPAD agar plates and imaged after 24 h incubation. A. P10-S1_CAP1/CAP1C446*, SC5314_CAP1/CAP1C446* and P10-S1_CAP1/CAP1C446* ::CAP1 were tested with wild-type strain SC5314 (CAP1/CAP1) as the control. White lines indicate the FLC MIC from Fig 2B. B. MEC079_CAP1/CAP1E448*, SC5314_CAP1/CAP1E448*and MEC079_CAP1/CAP1E448*::CAP1 were tested with wild-type strain (SC5314_CAP1/CAP1) as the control. White lines indicate FLC MIC from Fig 2B. C&D. Cell viability post-FLC MIC assay of (C) in vitro evolved mutant P10-S1_ CAP1/CAP1E446* and its mutated allele replaced strain (P10-S1_ CAP1/CAP1E446*::CAP1); (D) MEC079 _CAP1/CAP1E448* and its mutated allele replaced strain (MEC079 _CAP1/CAP1E448*::CAP1) from Fig 2B and S3 Data. Cell viability was determined by propidium iodide staining post 48 h MIC assay, together with all strains and wild-type control from Fig 2C (methods). Data were assessed for normality with a Shapiro–Wilk test, and significant differences between the CAP1 mutant and its mutated-allele replaced strain across different FLC concentrations were calculated using two-way ANOVA with Šídák’s multiple comparisons test (two-sided); * P < 0.05, ** P < 0.01, ns P > 0.05; the exact P values are **0.0015 and 0.0094, *0.0413 and 0.0119 for all indicated comparisons. Values are mean ± SD calculated from three biological replicates. The data underlying this Figure can be found in S3_Data. E. Growth rate (area under the curve, AUC, Y-axis) of CAP1 mutants with C-terminal truncation in the presence of different concentrations of H2O2 (0−10 mM, X-axis) and 4NQO (0–0.3 μg/ml, X-axis) with wild-type as the control. Data were assessed for normality with a Shapiro–Wilk test, and significant differences between CAP1 mutants and wild-type control across different 4NQO concentrations were calculated using two-way ANOVA with Dunnett’s multiple comparisons test (two-sided); *P < 0.05, ** P < 0.01; the exact P val [file pbio.3003631.s003.tif]

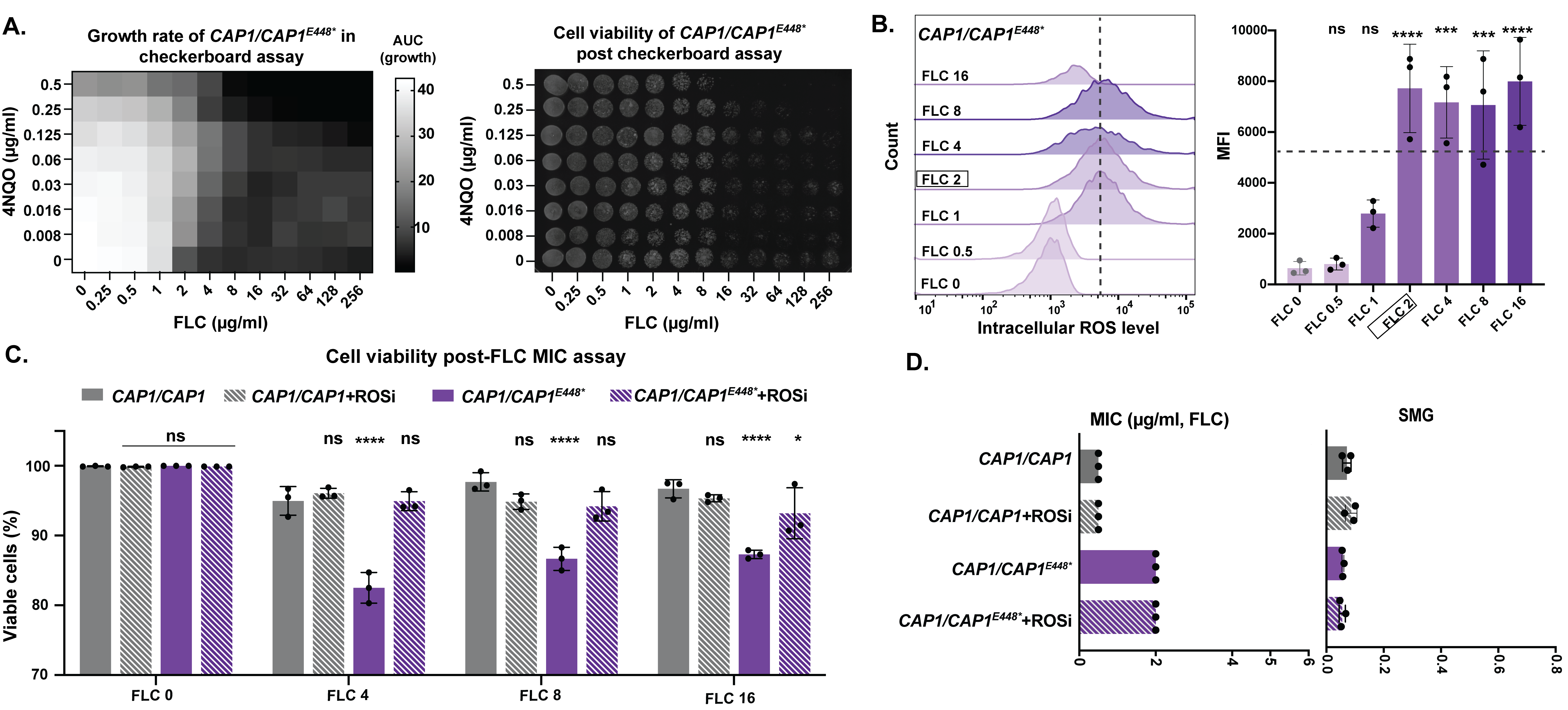

Supplement: S4 Fig — A. Checkerboard growth curve assay (left) and cell viability (right) of CAP1/CAP1E448* (SC5314 genetic background) in the presence of increasing concentrations of FLC (X-axis, 0–256 μg/ml, 2-fold dilutions) and/or increasing concentrations of 4NQO (Y-axis, 0–0.5 μg/ml, 2-fold dilutions). Growth rate was estimated with the area under the curve (AUC heatmap) of the 48 h growth curve. Cell viability: cells from growth curve were plated on YPAD agar and imaged after 24 h incubation. B. Intracellular ROS level of CAP1/CAP1E448* at different concentrations of FLC (FLC0-FLC16, μg/ml). Intracellular ROS was determined by ROS fluorescent detection kit combined with flow cytometry (methods). Left: Histogram of ROS fluorescent intensity from one representative biological replicate; Right: Median fluorescence intensity (MFI) of ROS and values are mean ± SD calculated from three biological replicates. Comparison was between FLC-exposed cells (FLC 0.5-FLC 16, μg/ml) and no-drug control (FLC 0) for each strain. Dotted lines indicate the highest level of intracellular ROS in the wild-type strain from Fig 3C. Black box indicates the MIC of test strains. C. The proportion of viable cells for wild-type (CAP1/CAP1) and CAP1/CAP1E448* post-FLC MIC assay at different concentrations of FLC (FLC 0-FLC 16) with or without ROS inhibitor (ROSi, S3_Data). Cell viability was determined by propidium iodide staining (methods). Statistical comparisons were against the FLC-only treated wild-type cells across different concentrations of FLC. The data underlying this Figure can be found in S3_Data. B&C: Data were assessed for normality with a Shapiro–Wilk test, and significant differences (B) one-way ANOVA with Dunnett’s multiple comparisons test and (C) two-way ANOVA with Dunnett’s multiple comparisons test (two-sided); ****P < 0.0001, * P < 0.05, ns P > 0.05; the exact P value is * 0.0226. D. 24 h MIC (left, μg/ml) and 48 h SMG (right) in FLC with or without ROSi treatment for CAP1/CAP1E448* with t [file pbio.3003631.s004.tif]

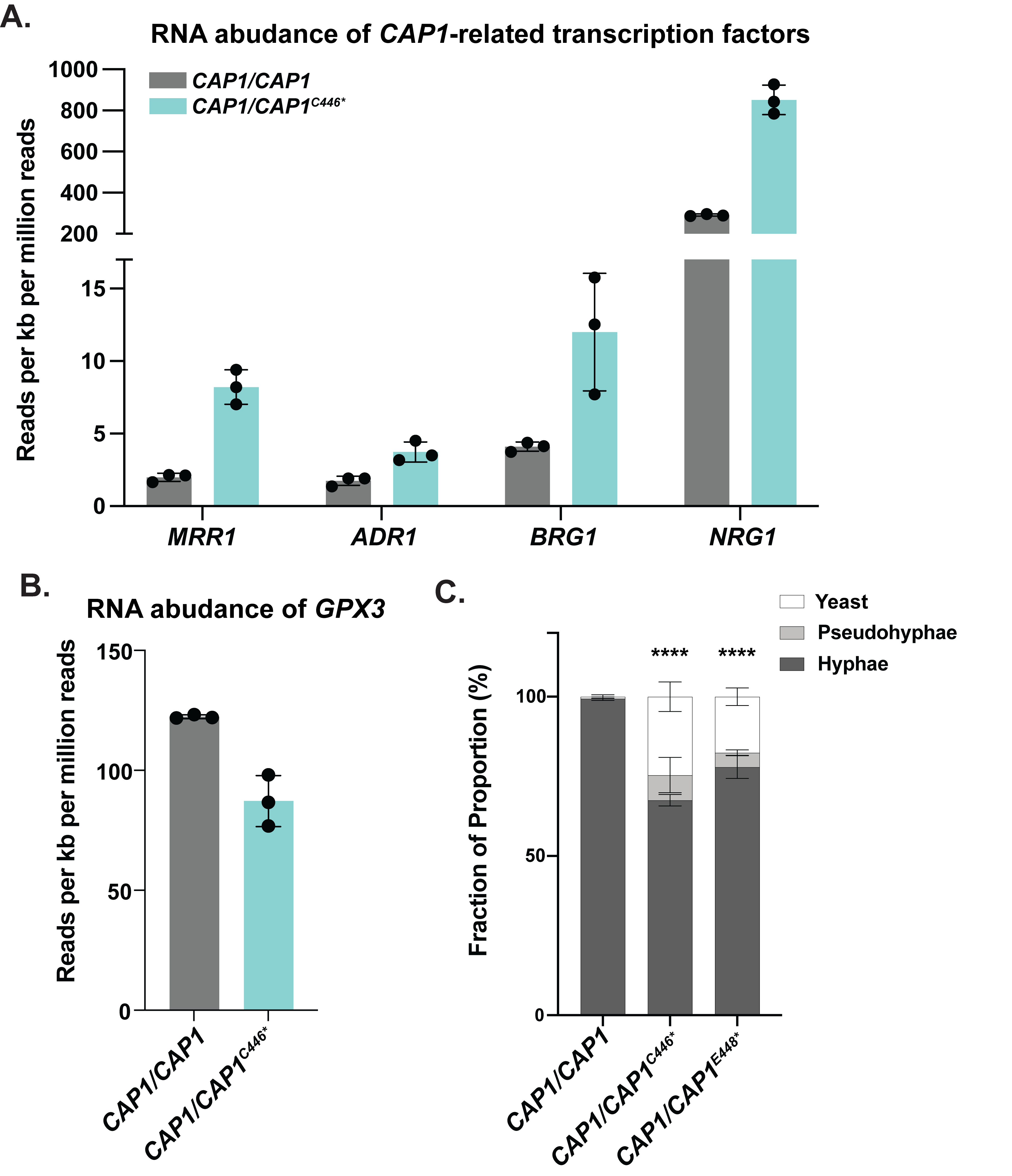

Supplement: S5 Fig — A&B: RNA abundance of CAP1-related transcription factors (A) and GPX3 (B) in wild-type strain (CAP1/CAP1) and CAP1/CAP1C446*. RNA reads were normalised to transcript length and total RNA reads. Values are mean ± SD calculated from three biological replicates. Each dot represents a single replicate. C. Quantification of the yeast (<6 μm), pseudohyphae (15–36 μm), and hyphae (>36 μm) for CAP1/CAP1C446* and CAP1/CAP1E448*with wild-type as the control. At least 100 cells were counted for each strain, and three biological replicates were performed. Values are mean ± SD calculated from three biological replicates. Statistical significance for filamentation was compared to CAP1/CAP1 and assessed using two-way ANOVA with Dunnett’s multiple comparisons test, ****P < 0.0001. The data underlying this Figure can be found in https://doi.org/10.5281/zenodo.18250101. (TIF) [file pbio.3003631.s005.tif]

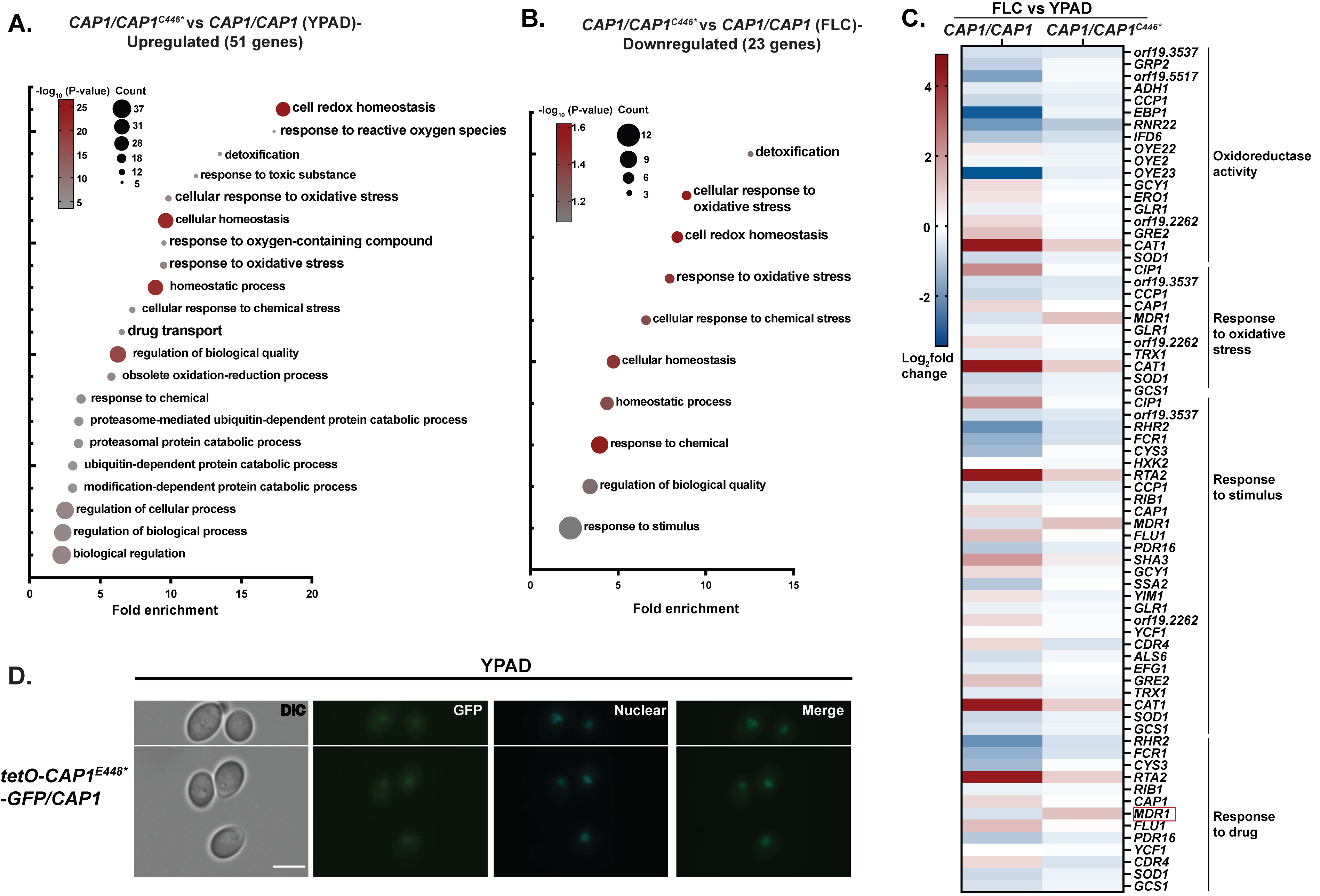

Supplement: S6 Fig — A. Gene Ontology (GO) terms for upregulated Cap1 targets in the CAP1/CAP1C446* mutant compared to CAP1/CAP1 in YPAD (S6_Data). The data underlying this Figure can be found in S6_Data. B. Gene Ontology (GO) terms for downregulated Cap1 targets in the CAP1/CAP1C446* mutant compared to CAP1/CAP1 in FLC (S8_Data). The data underlying this Figure can be found in S8 Data. A&B: X-axis: fold enrichment relative to the wild-type reference strain. Circle size indicates the gene counts of each term. The intensity of red indicates the adjusted P-values calculated by the Benjamini-Hochberg procedure. C. Expression changes (log2 fold change) of Cap1 target genes that are involved in oxidative stress response and drug transport in FLC relative to YPAD within wild-type (CAP1/CAP1) (left) and CAP1/CAP1C446* mutant (right) background. Gene ontology terms were adopted from [15]. The data underlying this Figure can be found in https://doi.org/10.5281/zenodo.18250101. D. Subcellular localization of Cap1 with E448* (TetO-CAP1E448*-GFP/CAP1), scale bar is 5 μm. Hoechst (light blue) was applied, indicating nuclear co-localization (Methods). (TIF) [file pbio.3003631.s006.tif]

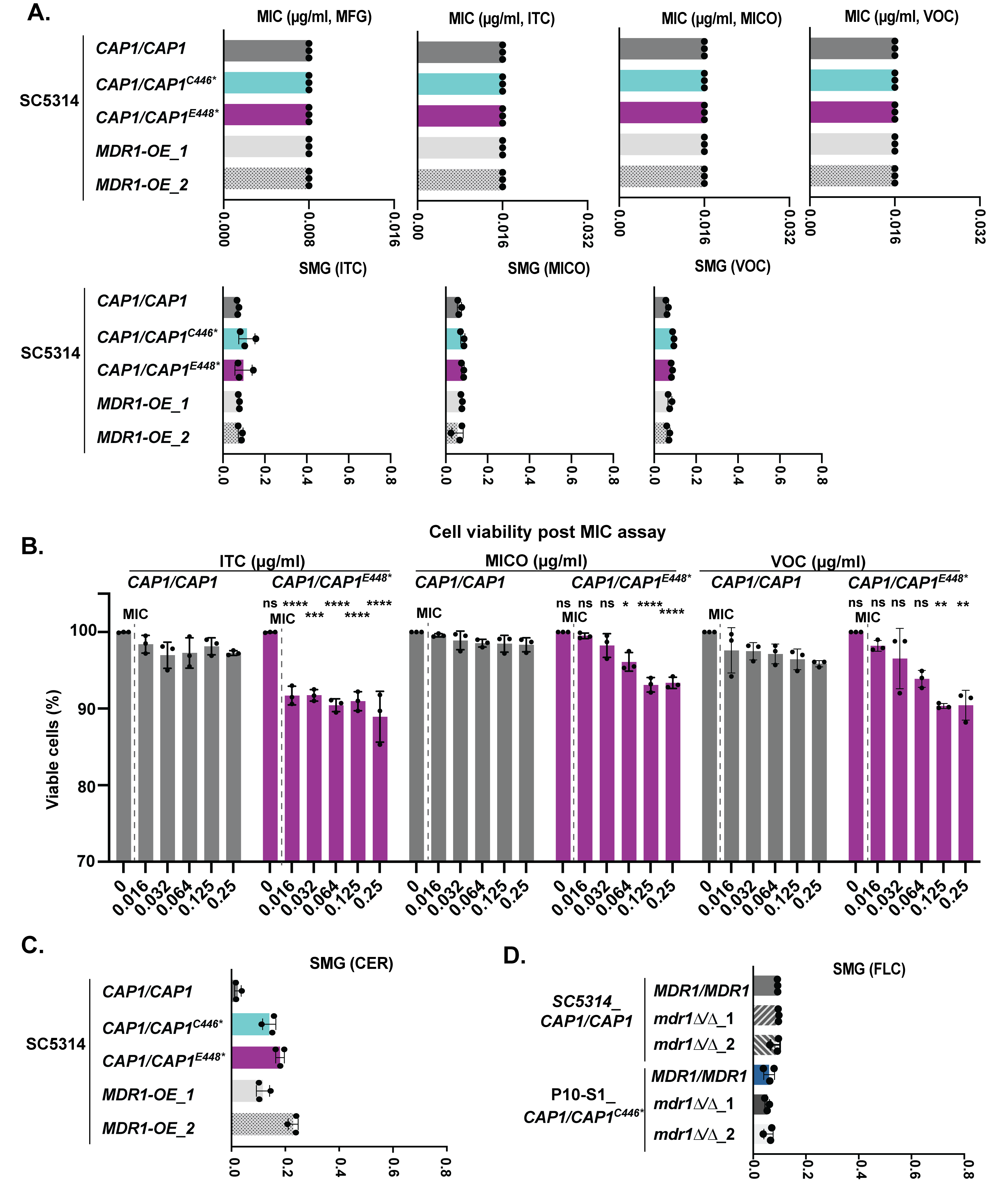

Supplement: S7 Fig — A. 24 h MIC (μg/ml) (Top) and 48 h SMG in micafungin (MFG), itraconazole (ITC), miconazole (MICO), and voriconazole (VOC) for CAP1/CAP1C446*, CAP1/CAP1E448*, and MDR1 overexpression strains MDR1-OE_1 and MDR1-OE_2, with wild-type background strain (SC5314, CAP1/CAP1) as the control. B. Cell viability post-FLC MIC assay. The proportion of viable cells for wild-type (CAP1/CAP1) and CAP1/CAP1E448* post 48 h MIC assay at different concentrations of ITC, MICO, and VOC (0–0.25 μg/ml). Cell viability was determined by propidium iodide staining (methods). Comparison was between CAP1/CAP1 and CAP1/CAP1E448* across different concentrations of drugs. Data were assessed for normality with a Shapiro–Wilk test, and significant differences using two-way ANOVA with Šídák’s multiple comparisons test (two-sided); ** P < 0.01,*** P < 0.001,**** P < 0.0001, ns P > 0.05; the exact P values are * 0.0144, ** 0.0012 and 0.0039, ***0.0010, and **** < 0.0001. C. 48 h SMG in cerulenin (CER) for strains CAP1/CAP1C446*, CAP1/CAP1E448* and MDR1 overexpression strains MDR1-OE_1 and MDR1-OE_2, with wild-type background strain (SC5314, CAP1/CAP1) as the control. D. 48 h SMG in FLC for FLC-evolved mutant P10-S1 (CAP1/CAP1C446*) and mdr1Δ/Δ_1 and _2 in P10_S1 background, with wild-type strain (CAP1/CAP1) and mdr1Δ/Δ_1&_2 in wild type background as the controls. Two independent transformants were included for MDR1 deletion mutants as the control. A&B&D: values are mean ± SD calculated from three biological replicates of a single strain and each dot represents a single replicate. The data underlying this Figure can be found in https://doi.org/10.5281/zenodo.18250101. (TIF) [file pbio.3003631.s007.tif]
