# Supplementary material for: Recurrent mutations in the stress regulator Cap1 reveal a trade-off between azole resistance and oxidative stress response in Candida albicans
Source: PLoS Biol. 2026 Feb 2;24(2):e3003631. doi: 10.1371/journal.pbio.3003631 (PMC12904577; doi:10.1371/journal.pbio.3003631)
Supplement: Attachment — Submitted filename: 2025_12_11_Response to Reviewers.docx [file pbio.3003631.s018.docx]

Dear Anna,

Thank you for your patience while your manuscript "Recurrent CAP1 mutations reveal a trade-off between drug resistance and oxidative stress response in Candida albicans" went through peer-review at PLOS Biology. Your manuscript has now been evaluated by the PLOS Biology editors, an Academic Editor with relevant expertise, and by three independent reviewers. My sincere apologies for the really long delay on the decision.

As you will see in the reports, all reviewers are positive about the study but give several suggestions that we will require you to address as we think they will make the manuscript stronger and clearer. Reviewer 1 suggests clarifying transcriptional comparisons between WT and truncation mutants, examining Cap1 stability using existing GFP/RFP fusions, and refining interpretation and presentation of nuclear-localization microscopy. Reviewer 2 requests a control (parental mdr1Δ/Δ strain in Fig 5D), and recommends clarifying the allele-replacement experiment in Fig 2B as well as adjusting the abstract to avoid implying that many clinical isolates carry similar truncations. Reviewer 3 recommends moving certain supplemental figures into the main figures and refining interpretation of the microscopy data.

In addition to these revisions, you may need to complete some formatting changes, which you will receive in a follow up email. A member of our team will be in touch with a set of requests shortly. If you do not receive a separate email within a few days, please assume that checks have been completed, and no additional changes are required.

We expect to receive your revised manuscript within 1 month. Please email us ([plosbiology@plos.org](mailto:plosbiology@plos.org)) if you have any questions or concerns, or would like to request an extension.

At this stage, your manuscript remains formally under active consideration at our journal; please notify us by email if you do not intend to submit a revision so that we withdraw the manuscript.

**IMPORTANT - SUBMITTING YOUR REVISION**
Your revisions should address the specific points made by each reviewer. Please submit the following files along with your revised manuscript:

1. A 'Response to Reviewers' file - this should detail your responses to the editorial requests, present a point-by-point response to all of the reviewers' comments, and indicate the changes made to the manuscript.
*NOTE: In your point-by-point response to the reviewers, please provide the full context of each review. Do not selectively quote paragraphs or sentences to reply to. The entire set of reviewer comments should be present in full and each specific point should be responded to individually.

You should also cite any additional relevant literature that has been published since the original submission and mention any additional citations in your response.

2. In addition to a clean copy of the manuscript, please also upload a 'track-changes' version of your manuscript that specifies the edits made. This should be uploaded as a "Revised Article with Changes Highlighted " file type.

*Resubmission Checklist*
When you are ready to resubmit your revised manuscript, please refer to this resubmission checklist: <https://plos.io/Biology_Checklist>

To submit a revised version of your manuscript, please go to <https://www.editorialmanager.com/pbiology/> and log in as an Author. Click the link labelled 'Submissions Needing Revision' where you will find your submission record.

Please make sure to read the following important policies and guidelines while preparing your revision:

*Published Peer Review*
Please note while forming your response, if your article is accepted, you may have the opportunity to make the peer review history publicly available. The record will include editor decision letters (with reviews) and your responses to reviewer comments. If eligible, we will contact you to opt in or out. Please see here for more details:
<https://blogs.plos.org/plos/2019/05/plos-journals-now-open-for-published-peer-review/>

*PLOS Data Policy*
Please note that as a condition of publication PLOS' data policy (<http://journals.plos.org/plosbiology/s/data-availability>) requires that you make available all data used to draw the conclusions arrived at in your manuscript. If you have not already done so, you must include any data used in your manuscript either in appropriate repositories, within the body of the manuscript, or as supporting information (N.B. this includes any numerical values that were used to generate graphs, histograms etc.). For an example see here: <http://www.plosbiology.org/article/info%3Adoi%2F10.1371%2Fjournal.pbio.1001908#s5>

*Blot and Gel Data Policy*
We require the original, uncropped and minimally adjusted images supporting all blot and gel results reported in an article's figures or Supporting Information files. We will require these files before a manuscript can be accepted so please prepare them now, if you have not already uploaded them. Please carefully read our guidelines for how to prepare and upload this data: <https://journals.plos.org/plosbiology/s/figures#loc-blot-and-gel-reporting-requirements>

*Protocols deposition*
To enhance the reproducibility of your results, we recommend that if applicable you deposit your laboratory protocols in [protocols.io](http://protocols.io/), where a protocol can be assigned its own identifier (DOI) such that it can be cited independently in the future. Additionally, PLOS ONE offers an option for publishing peer-reviewed Lab Protocol articles, which describe protocols hosted on [protocols.io](http://protocols.io/). Read more information on sharing protocols at <https://plos.org/protocols?utm_medium=editorial-email&utm_source=authorletters&utm_campaign=protocols> 

Thank you again for your submission to our journal. We hope that our editorial process has been constructive thus far, and we welcome your feedback at any time. Please don't hesitate to contact us if you have any questions or comments.

Sincerely,

Melissa

Melissa Vazquez Hernandez, Ph.D.
Associate Editor
PLOS Biology
[mvazquezhernandez@plos.org](mailto:mvazquezhernandez@plos.org)

----------------------------------------------------------------

REVIEWERS' COMMENTS

Specific responses to each reviewer comment are found below, in **blue font**.

----------------------------------------------------------------

Reviewer #1:

This study has identified acquired mutations in the Cap1 transcription factor that promote resistance of Candida albicans to sub-MIC concentrations of fluconazole, but intriguingly are more sensitive to supra-MIC fluconazole levels due to impaired oxidative stress responses. This provides novel insight into the relationship between antifungal drug and oxidative stress resistance, providing potential therapeutic strategies to offset drug resistance.

In this study heterozygous mutations in CAP1, resulting in the C-terminal truncation and removal of the nuclear export sequence from this transcription factor, were found following in vitro fluconazole evolution experiments (Cap1/Cap1C446*) and in a bloodstream isolate (Cap1/Cap1E448*). Convincing data is presented that both mutations increased the MIC of fluconazole. However, perhaps counterintuitively, despite increases in the MIC, strains expressing truncated Cap1 were more sensitive than wild-type cells to fluconazole concentrations above the MIC. Subsequent experiments revealed that at supra-MIC levels of fluconazole, the C-terminal truncated mutants accumulated higher levels of ROS than wild-type cells. This likely underpins the increased sensitivity of the C-terminal truncated mutants to fluconazole, as this could be reversed by addition of the antioxidant N-acetylcysteine. Gene expression analysis revealed that C-terminal truncation of Cap1 resulted in the activation of many Cap1 target genes including those involved in the oxidative stress response and drug transport, although this was impacted upon fluconazole treatment where several Cap1-dependent genes were down-regulated compared to wild-type cells. Subsequent mutational analysis indicated that upregulation of MDR1 contributed to the increased MIC to fluconazole (and cerulenin) in the Cap1 C-terminal truncated mutants. A final key finding was that the nonsense mutation at C446 conferred resistance to fluconazole in a mouse model of systemic disease.

Thank you for your appreciation of the work and the helpful, constructive feedback.

Below are a number of suggestions to potentially further improve the manuscript:

Considering the expression of Cap1 target genes, I'm wondering if different comparisons may give extra information regarding the transcriptional responses to fluconazole in Wt and Cap1/Cap1C446 cells. For example, it would appear that OSR genes are down-regulated in Cap1/Cap1C446 cells compared to wild-type cells following fluconazole treatment. However, could this be due to a significant increase in expression in Wt cells over the high but fixed 'basal' level of expression in Cap1/Cap1C446 cells? So rather than being downregulated the expression of OSR genes in the Cap1/Cap1C446 cells doesn't change with fluconazole, whereas a significant induction is seen in Wt cells? Calculating the relative fold induction in Wt -/ Wt + fluconazole, and in Cap1/Cap1C446 -/ Cap1/Cap1C446+ fluconazole would give information as to whether the magnitude of the response is impacted - ie is there any further induction of Cap1 target genes in Cap1/Cap1C446 cells in response to fluconazole, and how does the level of expression compare to that seen in wild-type cells?

Thank you for the thoughtful suggestion. The short answer is that indeed, the magnitude of the response to fluconazole is much stronger in wild-type cells than in the *CAP1/CAP1^C446*^* mutant cells. The conclusions drawn from Figure 4D are still correct (namely, that the oxidative stress response genes are downregulated in *CAP1/CAP1^C446*^* cells versus wild-type cells in the presence of fluconazole), however based on the Reviewer’s suggestion we now say that this is predominantly driven by the upregulation of the OSR in wild-type cells.

We added the following paragraph to the results section:

“To dissect the mechanism underlying the downregulation of the OSR in *CAP1* mutant cells, we quantified the relative fold induction of Cap1 gene targets in the mutant or wild-type cells, in response to FLC. The *CAP1/CAP1^C446*^* mutant exhibited a substantially more neutral transcriptional response of OSR genes compared to wild-type cells, in response to FLC (**new Fig S6C**). This finding indicates that the downregulation of OSR genes in the *CAP1* mutant was primarily due to its impaired ability to mount an OSR in response to FLC, in contrast to the robust induction of the OSR observed in wild-type cells. Additionally, *MDR1* which encodes a drug transporter, was induced further in the *CAP1/CAP1^C446*^* mutant in response to FLC, in contrast to no *MDR1* induction in the wild-type cells in response to FLC (**new Fig S6C**), suggesting that induction of this drug efflux pump was specific to the *CAP1*-truncation mutant.”

Using the GFP fusions did you examine the impact of the C-terminal truncations on Cap1 stability? Previous mutation of the c-CRD or truncation of Cap1 results in reduced protein levels (refs 15, 17), possibly through inhibiting association with Ybp1 which is also crucial for Cap1 stability (ref 24).

Thank you for this question and excellent suggestion to use existing constructs to monitor protein stability. We used an identical GFP reporter to tag the Cap1 protein, with or without a C-terminal truncation. We then utilized flow cytometry to quantify the Cap1-GFP fluorescence intensity as an indicator of Cap1 protein stability across >10,000 cells and three biological replicates. Under the constitutive promoter, both versions of the C-terminally truncated protein (Cap1^C446*^-GFP and Cap1^E448*^-GFP) had significantly decreased fluorescence intensity compared the wild-type Cap1-GFP, indicating that the C-terminal truncations caused decreased Cap1 stability. We added these data and results as **New Fig 5C**. This decrease in Cap1 stability is consistent with previous estimates quantified by Western blot (references 15, 17).

Notably, we also found that the two C-terminal truncated proteins were also significantly different from each other: the Cap1^E448*^-GFP had significantly decreased fluorescence intensity compared to the Cap1^C446*^-GFP (**New Fig 5C**). This indicates that the truncation at amino acid 448 results in less Cap1 stability than the truncation at amino acid 446, which might contribute to the phenotypic differences observed between these two truncation mutations *in vitro* and *in vivo.*

The unexpected finding in this study that azoles at supra-MIC concentrations exert fungicidal effects due to increased ROS accumulation in CAP1-C-terminal truncated mutants, shares some similarities to the unexpected synergistic killing of C. albicans observed following combinations of oxidative and osmotic stress. This synergistic killing was due to elevated levels of ROS which prevented Cap1 activity (ref 22). Whilst I think it is outside the scope of this work to look at post-translational modification of the C-terminal truncated Cap1 mutants to different fluconazole concentrations - some reference to the fact that increases in intracellular ROS have previously been shown to impact on Cap1 activation could be useful.

Thank you for the thoughtful and constructive comments. We agree that the fungicidal effects caused by azole-induced ROS accumulation in the Cap1 truncation mutants share similarities with the synergistic killing observed under the combination of oxidative and osmotic stress. Specifically, azole-induced ROS poses similar inhibition of oxidative stress transcriptional output on truncated Cap1 as the combination stress on wild-type Cap1.

Discussion of these similarities has now been added to the results section with relevant references (Kaloriti et al. 2014 and Kos et al. 2016)

We now state: “In the presence of fluconazole, the Cap1-dependent transcriptional regulatory network was disrupted, resulting in an upregulation of drug transporter expression and loss of an oxidative stress response. This azole-induced inhibition of Cap1 activity parallels previous findings where combined oxidative and osmotic stress led to intracellular ROS accumulation, which similarly suppressed Cap1 function, eliminated oxidative stress–responsive transcription, and ultimately caused fungal cell death (Kaloriti et al. 2014 and Kos et al. 2016).”

Minor points:
Figure 1C. It is not clear what Figure 1C shows with regard to the Cap1 mutation?

Thank you for the comment. The whole genome sequencing read depth analysis in previous Fig 1C was to highlight that the heterozygous *CAP1* mutants are euploid and do not have chromosome copy number changes, which is rare during *in vitro* evolution experiments in fluconazole. However, we agree with the reviewer that the *CAP1* mutation itself was not visible and whole genome sequencing copy number analysis was included more as a control experiment. Therefore, we moved the previous Fig 1C to the **new Fig S1**, where we added a new sub-panel showing the Illumina sequencing reads that support the heterozygous *CAP1* variants in three single colonies sequenced at passage 10 of the evolution experiment in fluconazole.

Line 217. The conclusion that high concentrations of FLC are fungicidal to cells carrying a CAP1 C-terminal truncation does not hold true for the MEC079 Cap1/Cap1E448* mutant (Figure S3C). Also legend for Fig 2C needs clarifying in that only the C446* mutants were examined.

Thank you for these comments that additional clarification was needed. We agree that the MEC079 clinical isolate (MEC079_*CAP1/CAP1^E448*^*) did not exhibit decreased viability at high concentrations of fluconazole relative to SC5314. However, the same mutation engineered into the SC5314 genetic background (SC5314_ *CAP1/CAP1^E448*^)* was fungicidal at high concentrations of fluconazole (**updated Fig 2C**).

We attribute the survival of MEC079 at high concentrations of fluconazole to one or more of the ~11,000 SNP differences between SC5314 and MEC079 (more details below). Because of these genetic differences, the allele replacement experiment that we performed with MEC079 to replace the mutated allele with the wild-type allele (MEC079_*CAP1/CAP1^E448*^::CAP1*) provided additional support that Cap1 has a small role in viability in this strain background. The allele-replacement strain had significantly increased viability relative to MEC079 at 4 ug/ml fluconazole (Fig S3C). Because viability of MEC079 was already high, the sensitivity of this assay was limited.

For additional clarification regarding genetic backgrounds, we reorganized both figures Fig 2C and Fig S3C to improve the distinction between genetic backgrounds and their complemented/allele replacement strains. Additionally, the revised figures enable direct comparison between the C446* and E448* mutations, independently engineered in the SC5314 genetic background. **Updated Fig 2C** now highlights the mutants engineered in the SC5314 genetic background, including both *CAP1* mutants (*CAP1/CAP1^C446*^* and *CAP1/CAP1^E448*^*). Both C446* and E448* engineered mutants cause fluconazole to become fungicidal at high concentrations of fluconazole (Fig 2C).

**Updated Fig S3** contains the viability assay for genetic backgrounds that underwent allele-replacement:

**Updated Fig S3C** now includes the *in-vitro* evolved *CAP1* mutant

(P10-S1_*CAP1/CAP1^C446*^*) and its mutated allele replaced strain

(P10-S1_*CAP1/CAP1^C446*^::CAP1*).

**Updated Fig S3D** now includes data for the MEC079 clinical isolate (MEC079_*CAP1/CAP1^E448*^*) and its mutated allele replaced strain MEC079_*CAP1/CAP1^E448*^::CAP1*).

Additionally, we performed variant analysis on MEC079 and identified approximately 11,000 additional variants compared to the wild-type SC5314. Including ~100 nonsynonymous variants within Cap1 target genes including *CIP1, GCS1* and *GLR1* which are related to the oxidative stress response. We hypothesize that other genetic variants in MEC079 rescue the viability defect in this genetic background.

We revised the results section, and the updated figure legends accordingly. The results section now reads: “However, no decrease in viability was observed for clinical isolate MEC079 relative to SC5314 or its mutated allele replaced strain (Fig S3B&D), yet the same mutation engineered into the SC5314 (SC5314_ *CAP1/CAP1^E448*^)* was fungicidal at high concentrations of fluconazole. We attribute the survival of MEC079 at high concentrations of fluconazole to one or more of the ~11,000 SNP differences (allele frequency >0.3) between SC5314 and MEC079, including ~100 nonsynonymous variants within Cap1 gene targets that are related to oxidative stress response (e.g., *CIP1, GCS1* and *GLR1*). These viability results suggests that high concentrations of FLC are fungicidal to SC5314 cells carrying a *CAP1* C-terminal truncation, while clinical isolate MEC079 has acquired additional sequence variants that appear to rescue this killing effect.”

Figure S6 spelling of nuclear

Thank you for catching this. The spelling has been fixed.

Figure 4E - the CAP1-RFP fusion appears to form discrete loci (not nuclear) rather than being cytoplasmic?

Thank you for this thoughtful observation. To address this and other Reviewer comments, we did a more careful analysis of the RFP fluorescence images. As a reminder, the RFP in these experiments tagged the wild-type allele of *CAP1*. We conclude that the wild-type *CAP1-RFP* in the *CAP1^C446*^/CAP1-RFP* background exhibited punctate subcellular localization adjacent to the nucleus in YPAD (**New Fig 5A**).

We updated the results section to read: “The mutated Cap1 protein also influenced the subcellular localization of wild-type Cap1 protein. In YPAD, the fluorescently tagged wild-type Cap1 protein exhibited punctate localization adjacent to the nucleus in the *CAP1^C446*^/CAP1-RFP* cells (Fig 5A, RFP), but diffuse cytoplasmic distribution in the *CAP1-GFP/CAP1* wild-type cells.”

Figure 6. Can the authors share their thoughts why the patient derived Cap1E448 mutant does not result in drug resistance in vivo in contrast to Cap1C446.

Thank you for this important question. We believe that several factors contribute to the different drug susceptibility patterns observed *in vivo* between the C446* and E448* mutations (Fig 7A). As a reminder, the difference *in vivo* was consistent with our *in vitro* observations, where the *CAP1/CAP1^C446*^* mutant exhibited a 2-fold higher FLC MIC compared to *CAP1/CAP1^E448*^* when both were engineered into the same genetic background (SC5314). In the murine model of infection, the difference in drug susceptibility between the mutants was exasperated.

We propose the following mechanistic explanation: truncation at C446 completely abolishes disulfide bond formation and maintains Cap1 in a constitutively active state that is independent of oxidation. In contrast, truncation at E448 might still permit disulfide bond formation, however at substantially reduced efficiency, which paradoxically interferes with Cap1 activation in the absence of oxidative stress. This would result in reduced transcription of drug transporters and reduced resistance to drug stress in cells with E448 truncation relative to cell with C446 truncation.

We also performed a new RT-PCR experiment to determine the mechanism underling the observed phenotypic differences. The results now read: “…we applied RT-PCR to compare the transcriptional level of major drug transporter *MDR1* and *CAP1* in cells with truncation at C446 or E448 cultured *in vitro*. Both mutants had elevated expression of both *MDR1* and *CAP1* relative to wild-type. The mutant with the C446 truncation had ~1.5-fold higher *MDR1* expression than cells with the E448 truncation (5.2-fold and 3.5-fold respectively **new Fig 7B**), which likely contributes to the difference observed between the two truncation mutations *in vivo*”.

Additionally, as we described above, protein stability was significantly different between the two C-terminal truncation mutants. Cap1^E448*^-GFP had significantly decreased fluorescence intensity compared to the Cap1^C446*^-GFP (**New Fig 5C**). This indicates that the truncation at residue 448 results in decreased Cap1 stability compared to the truncation at residue 446, and this stability might be exasperated *in vivo*.

We updated the results and discussion text accordingly.

----------------------------------------------------------------

Reviewer #2:

This is a very interesting paper from the Selmecki group exploring the role of mutations in CAP1 in fluconazole resistance. They demonstrate that c-terminal truncation of Cap1 results in constitutive activation and an increase in fluconazole MIC but at the cost of increased cell death at supra-MIC levels of fluconazole or other azole drugs. I largely found the claims made here to be well supported, although there is one case where I'd like to see an additional control (below). I also found one line in the abstract to be slightly misleading. The authors mention identifying 300 additional clinical isolates and identifying 25 distinct CAP1 missense or nonsense alleles. Prior to reading the text, I took this to mean there were many additional clinical cases with similar phenotypes to the Cap1 truncation allele. Rather, there is exactly one experimentally evolved line and one clinical isolate with this genotype/phenotype and no others with truncations in similar locations. I think reordering the abstract a little would alleviate this issue for me (suggestion below). Neither of these issues significantly dampened my enthusiasm for the story presented here. The somewhat paradoxical cost of the increased resistance is quite interesting and the authors performed a convincing set of experiments to tie this phenotype to redox stress. I think this will be of broad interest to the community working on antifungal drug resistance.

Thank you for your appreciation of the work and helpful feedback.


Major comments:

I am a little confused by the mechanism of Cap1 truncation resistance as depicted in Figure 2B. The authors propose that this is the result of a c-terminal truncation that results in increased activity of Cap1 by eliminating the nuclear export signal and causing constitutive activation of Cap1's client genes. I would predict this mechanism to result in a dominant phenotype for the Cap1 truncation alleles, especially given that it is conferring resistance as a heterozygous allele. However, Figure 2B shows complementation of this allele via addition of another copy of Cap1 that returns it to a wildtype phenotype. I think perhaps this experiment is actually an allele replacement of the truncation allele, but it is not clear from the text in the results or the genotype in the figure. If so, describing this more clearly and altering the genotype to reflect what is actually happening in this experiment would help.

We regret that Figure 2B was confusing and appreciate that clarification was needed. Indeed, we performed allele replacement of the truncated allele with the wild-type *CAP1* allele. We now use *CAP1/CAP1^E448*^::CAP1* to clearly indicate that the *CAP1^E448*^* allele was replaced with the wild-type *CAP1* allele. We have updated the manuscript text and genotypes in the figures accordingly.

Lines 16-17 in the abstract come out of order with the order told in the main text. I would move this up to line 5. As it stands this led me to expect an expanded story including many strains with similar phenotypes, although that case is not made here.

Thank you for the suggestion. We moved the suggested line to earlier in the abstract.

Figure 5D would benefit from a parental control mdr1 double deletion. The authors use this data to make the case that mdr1 overexpression is responsible for the increase in resistance in the Cap1 truncation alleles. I suspect they are right but without seeing what the null looks like, it is hard to distinguish this hypothesis from the competing hypothesis that mdr1 nulls simply decrease resistance in all contexts.

Thank you for the constructive suggestion. We generated two independent *mdr1* null transformants in the SC5314 *CAP1/CAP1* background and quantified fluconazole MIC and tolerance (**New Fig 6D & Fig S7D**). These new *mdr1* null controls provide direct comparison between the *mdr1* null mutation in the *CAP1/CAP1^C446*^* background. Deletion of *MDR1* in the SC5314 *CAP1/CAP1* background had no impact on FLC susceptibility (MIC or tolerance), whereas deletion of *MDR1* in the *CAP1/CAP1^C446*^* mutant resulted in a 4-fold decrease in the fluconazole MIC (**New Fig 6D)**. These results support that upregulation of *MDR1* in *CAP1* mutants is responsible for the increased MIC.

Minor Comments:
There are a number of spelling mistakes throughout the figures, some of which are documented below. I would take a thorough editing pass through the figures, checking for spelling.

Thank you for catching these typos. We have reviewed all figures and corrected the spelling errors throughout the manuscript. Specifically: viability in Fig 2C, 3E, 6C, S3C, S4C, and S7B and nuclear in Fig 5A&B and S6D.

Fig 1C: I wish there was a more visual way of depicting that these were CAP1/CAP1C446* hets in the figure here. Perhaps an inset on the CAP1 locus? The figure legend here also does not explain what a white background means in the figure (I'm assuming no diversity to score heterozygosity?).

Thank you. To address this and other Reviewer comments, we moved the original Fig 1C from the main text into the supplement (**New Fig S1A**) and added a new figure showing a zoomed in view of the heterozygous *CAP1* locus from the whole genome sequencing data (**New Fig S1B**). The legend was updated for clarity regarding all colors and mapping tools utilized to generate the figure.

Fig 2C: Viability is spelled incorrectly in the title

Thank you for catching this. The spelling has been fixed.

Fig 2C: I think this assay would benefit from another set of experiments at 1 ug/mL that would show whether viability defects begin above the MIC or below.

Thank you for the suggestion. In the initial submission, we included 0, 0.5, 1, 2, 4, 8, 16 μg/ml FLC, for a total of 7 different fluconazole concentrations in the supplementary Table S3. We agree that the data for 1 μg/ml is important to highlight that the viability defect begins above the MIC and have **updated Fig 2C** to include these data.

Line 243-244: Opening phrase of this sentence is not quite grammatically correct

Thank you for pointing out this. Grammar issues have been fixed.

Fig 3E: Viability spelled incorrectly again

The spelling has been fixed.
Figure S3: Viability spelled incorrectly in a few ways here

The spelling has been fixed.
Figure S6 nuclear is spelled incorrectly

The spelling has been fixed.


----------------------------------------------------------------

Reviewer #3: PBIOLOGY-D-25-03189R1

This manuscript characterizes the CAP1 mutations that confer azole resistance in clinical isolates of Candida albicans. Understanding mechanisms of drug resistance is important to understand how to improve management and treatment of candidiasis in the face of growing azole resistance. The work presented here is the first to characterize mutations of CAP1 in clinical isolates and their role in fitness, drug resistance and the oxidative stress response. This is a well-designed and robust study with a few minor comments to address prior to publication:

We appreciate your positive comments and suggestions for improvement.

1.      Figure 1C is hard to read/see colors; I think this figure would fit better in the supplemental figures while Supplemental Figure 1 should be moved to the main figures

Thank you for the thoughtful and constructive suggestions. To address this and other Reviewer feedback, we moved Fig 1C into a **new Fig S1** and the previous Fig S1 to the main text (**new Fig 2A**). New Fig 2A now contains the variants identified from both clinical isolates (top) and the *in vitro* evolution experiment (bottom) with nonsense mutations highlighted with red box.

2.      Similarly, I think the fluconazole treated cells for nuclear localization microscopy should be included in the main figure.  I don't necessarily agree with the interpretation of the nuclear localization microscopy results.  In the fluconazole treated samples (Fig S6D) wild type CAP1 does maintain diffuse cytoplasmic staining but there are a few clear puncta that localize with the nucleus unlike in the YPD samples where it is truly diffuse. However, the Cap1C446* treated with fluconazole look more like the CAP1-RFP (Figure 4E) where staining is a diffuse-puncta that is adjacent to the nucleus but does not overlap. Even in the CAP1-C446*-GFP images in figure 4E, there are bright puncta that overlap with the nuclei that are also associated with a diffuse staining adjacent to the nucleus.  I agree that there are differences between the WT and mutant in YPD vs FLC, however it is not as simple as either being in the nucleus or diffuse cytoplasmic staining.

Thank you for the constructive feedback. First, we moved the fluconazole-treated microscopy images to the main figure (**new Fig 5B**). Second, we appreciate that Cap1 localization needed a more nuanced description, as the fluorescence is not as simple as being either in the nucleus or cytoplasm. We updated the results as follows:

We describe the subcellular localization changes of the wild-type *CAP1* and *CAP1^C446*^* upon fluconazole exposure as follows: “FLC exposure induced abnormal nuclear and cell morphologies, which interfered somewhat with the nuclear localization of the truncated Cap1 protein, resulting in its accumulation adjacent to punctate nuclei. In contrast, the wild-type Cap1 protein in wild-type cells mostly maintained its diffuse cytoplasmic localization with a few puncta that seem to colocalize with nuclei (Fig 5B).”

Additionally, we added a more detailed description of the localization of the wild-type *CAP1-RFP* in *CAP1/CAP1^C446*^* background: “The mutated Cap1 protein also influenced the subcellular localization of wild-type Cap1. In YPAD, the fluorescently tagged wild-type Cap1 protein exhibited punctate localization adjacent to the nucleus in the *CAP1^C446*^/CAP1-RFP* cells (Fig 5A, RFP), but diffuse cytoplasmic distribution in the *CAP1-GFP/CAP1* wild-type cells (Fig 5A, GFP top panel).
